# Patient and Clinician Perspectives of Physical Therapy for Walking Difficulties in Multiple Sclerosis

**DOI:** 10.1155/2023/1121051

**Published:** 2023-02-15

**Authors:** Prudence Plummer, Andrea Stewart, Jessica N. Anderson

**Affiliations:** ^1^Department of Physical Therapy, MGH Institute of Health Professions, Boston, MA 02129, USA; ^2^Adult Inpatient Division, Department of Physical Therapy and Occupational Therapy, Duke University Health System, Durham, NC 27710, USA; ^3^University of North Carolina Hospitals, Department of Rehabilitation Therapies, Chapel Hill, NC 27514, USA

## Abstract

Gait speed is frequently the primary efficacy endpoint in clinical trials of interventions targeting mobility in people with multiple sclerosis (MS). However, it is unclear whether increased gait speed is a meaningful outcome for people living with MS. The purpose of this study was to identify the most important aspects of mobility for people with MS and physical therapists and to explore how patients and clinicians perceive whether physical therapy has been effective. Forty-six people with MS and 23 physical therapy clinicians participated in a focus group, one-on-one interview, or electronic survey. The focus group and interview data were transcribed and coded to identify themes. Free-text survey responses were also coded, and multiple-choice options were analyzed for frequency. Among people with MS, falls and difficulties getting out into the community were identified as highly important mobility limitations. Clinicians also identified falls and safety as a priority. Walking speed was infrequently described as a problem, and although gait speed is often measured by clinicians, improving gait speed is rarely a treatment goal. Despite their emphasis on safety, clinicians lacked certainty about how to objectively measure improvements in safety. People with MS evaluated physical therapy effectiveness based on the ease by which they can do things and acknowledged that “not getting worse” is a positive outcome. Clinicians evaluated effectiveness based on the amount of change in objective outcome measures and by patient and caregiver reports of improved function. These findings indicate that gait speed is not of major importance to people with MS or physical therapy clinicians. People with MS want to be able to walk further and without an assistive device, and they want to avoid falls. Clinicians want to maximize safety while improving functional ability. Clinicians and patients may differ in their expected outcomes from physical therapy.

## 1. Introduction

One of the most frequent symptoms of multiple sclerosis (MS) is difficulty walking. Mobility limitations affect more than 90% of people with MS [1], and of those who experience difficulty walking, 70% consider it to be the most challenging aspect of living with MS [2]. The reason that walking impairments may have such a devastating impact on the lives of people with MS may be due to the fact that the disease onset typically occurs in people 20-40 years old [3], which is much earlier in life than the average age of onset of other neurodegenerative diseases (e.g., Parkinson's disease and amyotrophic lateral sclerosis). Thus, the potential impact of walking limitations on social and work life, recreational activity, the ability to travel, and to perform activities of daily living in people with MS is substantial [2, 4–6]. Indeed, impaired mobility is one of the two main reasons (second to fatigue) for job loss in people with MS [7]. It is not surprising, therefore, that walking ability is one of the top priorities for individuals with MS [8].

The most common intervention approaches for treating mobility limitations in people with MS are rehabilitation (such as physical therapy or gait training), exercise training, and dalfampridine (known as fampridine outside the United States). Gait speed is frequently the primary endpoint in studies to evaluate the efficacy of these interventions [9–13]. Despite apparent efficacy of many of these intervention approaches based on improvements in gait speed, it is not clear whether people living with MS consider gait speed an important improvement in functional mobility. Thus, the meaningfulness of therapeutic efficacy from the patient perspective for these various intervention approaches is largely unknown. It is also not known how rehabilitation clinicians make decisions about therapeutic effectiveness in the management of gait difficulties in people with MS. It is imperative to understand which aspects of mobility are considered most important to people living with MS to improve the design and potential impact of future rehabilitation studies.

The purpose of this qualitative study was to identify the most important aspects of mobility for people living with MS as well as physical therapists who treat people with MS. A secondary focus was to explore how patients and clinicians perceive whether physical therapy has been effective.

## 2. Materials and Methods

This qualitative study was completed in two stages. Firstly, we conducted a series of focus groups and one-on-one interviews separately for people with MS and for physical therapy clinicians with experience treating individuals with MS. The findings from the focus groups and interviews were then used to develop an electronic survey that could be distributed more broadly to patient and clinician stakeholders. Focus group and interview participants were recruited purposively through flyers distributed to professional groups and to local MS self-help groups. For the electronic survey, the invitation and link to access the survey was distributed via email to professional associates of the researchers and to the membership of the local chapter of the National MS Society. The associates of the researchers were also asked to share the survey invitation with their patients with MS and professional colleagues. The study was approved by the Institutional Review Board of the University of North Carolina at Chapel Hill. All participants provided informed consent to participate.

Forty-six people with MS and 23 physical therapy clinicians participated. Of the 46 participants with MS, 4 participated in a focus group, 1 participated in a phone interview, and 41 responded to the electronic survey. Of the 23 physical therapists, 6 participated in a focus group, 1 participated in a phone interview, and 16 responded to the electronic survey. Initially, a greater number of focus groups was planned but lack of geographical proximity and mutual availability of interested participants restricted further focus groups from being conducted. Thus, as an alternative, individuals who had been unable to join a focus group were invited to complete the electronic survey or a one-on-one a phone interview. There are no rules governing the optimal sample size in focus group research; rather, the number of groups is determined by the objectives of the research and saturation of the data [14]. Triangulation of the focus groups, interviews, and online survey served the objectives of this research.

### 2.1. Focus Groups and Interviews

The focus groups were moderated by the first author, who is experienced in the focus group methodology [15, 16]. An assistant moderator and a notetaker (AS and JA) were also present in each focus group to monitor the recording equipment and take notes on the discussion, especially the nonverbal behavior, to supplement the transcripts and aid the analysis. Focus group participants, including the moderator, assistant moderator, and notetaker, were seated in a circular formation, as recommended to facilitate discussion between participants [15]. The moderator also conducted the one-on-one phone interviews, which were similarly recorded for verbatim transcription. The question routes for the focus groups were developed in advance specifically for the objectives of this study and were based on the triangular structure proposed by Hurworth [17]. That is, they began with introductory questions to introduce the topic and stimulate discussion, followed by transition questions designed to steer the participants to key questions of interest [15, 17]. Introductory questions asked participants with MS to describe their experiences with physical therapy and clinicians were asked to describe their experiences of treating people with MS. Transition questions then asked people with MS to discuss the difficulties they experience with walking, how their mobility limitations impact their daily life, and more details about their specific experiences with physical therapy, including what they found most and least effective. Transition questions for physical therapists also asked about the walking problems experienced by people with MS and the types of treatment activities they use to address mobility limitations. Finally, for people with MS, the key questions asked how they knew if a treatment had been effective and what had to improve or change to feel like they had benefited from physical therapy. People with MS were also asked what they considered most important when choosing a treatment option (e.g., cost and expected benefit). Key questions for clinicians asked about the assessment tools used to measure and document mobility difficulties and to evaluate treatment effects and how they determined if treatment had been effective. The one-on-one interview question schedules followed the same structure as the focus groups.

### 2.2. Electronic Survey

The surveys were developed using Qualtrics software (Qualtrics, Provo, UT). The responses were completely anonymous. After consenting to participate in the survey, participants were directed to a series of questions that generally followed the structure and questions of the focus groups and interviews. The question types included multiple-response options (e.g., select all that apply) and open-ended questions. The focus groups and interviews helped inform the response options, but survey participants were always provided with an option to write in their own response. Demographic information was requested at the end of the surveys to help describe the study samples.

### 2.3. Analyses of Data

The focus groups and interviews were transcribed verbatim by a professional transcription service (Rev, San Francisco, CA). The transcripts were then edited to remove any names and identifiable information about the participants. Focus group transcripts were further edited to add information about nonverbal behavior and group dynamics from the field notes taken during the discussions, as well as document the seating arrangement. Each transcript was read several times by each author independently, and potential themes were noted. The major ideas in response to each question, along with sample quotes, were then organized into a matrix to further assist in the identification of themes. An audit trail was also constructed in the data matrix to identify the origin and context of the data segments so that the researchers could retrace the data to its location in the transcript. The surveys were analyzed for frequency of response for multiple-choice options. Free-text answers were examined for themes, similar to the transcripts.

The trustworthiness of the findings was maximized by several methodological strategies that enhance the dependability, credibility, transferability, and confirmability of the data [16]. Specifically, use of the same question route as well as the same moderator/interviewer for each focus group and interview increases the rigor of the study [18]. Purposive sampling ensured that only physical therapists interested in the topic were sampled [19]. Transferability is enhanced by providing a detailed description of the patient and clinician samples and presenting the findings as much as possible in the words of the participants [16]. Finally, the construction of an audit trail to trace the data back to their origin as well as use of different methods to collect and triangulate the data further improves the credibility, confirmability, and dependability of the findings [16, 20]. In the results, the source of the data is indicated in parentheses following direct quotes or transcript excerpts (focus group (FG), interview, or survey), with “MS” indicating that the comment came from a person with MS and “PT” indicating that the comment came from a physical therapist.

## 3. Results

### 3.1. Description of Participants

The 46 participants with MS mostly had relapsing-remitting MS, but individuals with primary or secondary progressive MS also participated. In addition, 3 of the survey respondents with MS reported having radiologically isolated syndrome. Disease duration ranged from 1 to 30 years and at least 65% (*n* = 25) were currently using an assistive device to walk or mobilize (e.g., cane); 10 survey respondents did not provide a response to the assistive device question. The 23 physical therapists ranged in experience from less than 1 year to 40 years and represented a variety of practice settings, including acute care, inpatient and outpatient rehabilitation, home health, and school-based physical therapy. Seven therapists (30%) were certified neurologic clinical specialists by the American Board of Physical Therapy Specialties. The majority of participants in each cohort were female, which is consistent with the sex distribution among physical therapists in the United States [21] and people with MS [22].

### 3.2. Walking Difficulties

In describing their walking difficulties, two major themes emerged for people with MS: falls and difficulties getting out into the community. For example, one focus group participant with MS commented that, “I've stopped going out a lot. I don't go out every day and walk like I used to.” It was clear that not being able to go out easily or often has a broader impact on socialization as well as participation in life roles and recreation activities, as exemplified by the following statements:

“It's the getting out, the social.” (FG-MS).

“I can't really go to the store… I can't take our dog on a walk unless I'm in the electric wheelchair because I can't walk that far without feeling like I'm actually falling.” (interview-MS).

“Gardening is a problem. I mean, I feel like a lot of the things I used to do have really been, you know, I've really kind of had to give up a lot of those things.” (Interview-MS).

Limitations in going out were sometimes related to difficulties with transportation:

“I don't get out as much, like I'm driving my daughter's car more than getting on the bus. Because getting on the bus, I have to step up ‘cause some of these bus drivers won't put down the ramp for me to roll up (my walker).” (FG-MS).

Others were concerned about being dependent on someone else to assist them when they go out, for example, “to push me in the portable wheelchair.” (Interview-MS).

Slow walking speed was mentioned as a walking-related problem for people with MS, but mainly in the context of having to “take my time” (FG-MS) because of the time and energy required to walk or to avoid falling while walking. Indeed, it was clear that walking can be quite attention-demanding, “like babies when they start walking” (FG-MS), and that walking slowly is often a deliberate adaptive strategy to maintain safety or to conserve energy. More important than walking difficulties for some people with MS was not being able to get up from a fall:

“I mean, my walking sucks, okay. But I get around. (Other participants nod in affirmation.) I use a rollator at home and this (scooter) I use for distances and energy conservation. But if I fall in my house, there are times I've dialled 9-1-1 and just said, ‘This is not an emergency, I just need someone to come and pick me up (off the floor).'” (FG-MS).

The survey results from people with MS (*n* = 41) corroborated the importance of falls as a key mobility concern and that walking speed was not viewed as one of the most important walking difficulties. When identifying difficulties that limit walking, the top 5 selected survey responses were as follows: I fall or lose my balance (*n* = 34, 83%), I have difficulty with stairs (*n* = 33, 80%), I get very fatigued with walking (*n* = 29, 71%), I drag my foot on the ground when I walk (*n* = 27, 66%), and walking takes up so much energy (*n* = 26, 63%). Walking slowly/unable to walk quickly (*n* = 24, 59%) followed difficulties with knee buckling (*n* = 25, 61%) and poor sensation or presence of tingling (*n* = 25, 61%) were also common responses.

The findings from the physical therapists were consistent with those of the people living with MS in that falls were identified as the most frequent major problem related to mobility (75% survey respondents and all focus group and interview participants). Clinicians also reported that fatigue, dynamic balance, and weakness, especially in the lower extremity, including foot drop, were key problems. The theme of getting out into the community also arose in the discussions with clinicians:

“Just not being able to walk in stores and things like that… I mean, they tell you they can't do what they'd like to do in the community.” (FG-PT).

Regarding the importance of walking speed, the physical therapists said, “it depends on what they need to function” (FG-PT) as well as “the job that they're working and their responsibilities as an adult” (interview-PT) and whether it is “an applicable functional goal” (survey-PT). However, there was widespread agreement that “the first priority is to be able to walk safely” (FG-PT). One physical therapist commented that, “(The patient) may say, ‘I'm having a hard time with walking' or ‘I'm falling a lot,' but I've never heard someone say, ‘I can't walk quick enough.'” (FG-PT). Only 4 (25%) clinician survey respondents selected “slow gait speed” as a major problem for people with MS.

It was recognized by the clinicians that being able to walk quickly was necessary “to be safe in the community” (interview-PT) such as “to cross a street” (Survey-PT) or “avoiding traffic” (survey-PT). Clinicians were also concerned about a patient's safety at faster walking speeds and whether “we are improving gait speed at the cost of better gait mechanics” (survey-PT).

In line with the major themes from the people living with MS, clinicians also stated that not falling, “walking better” (FG-PT) or “(walking) further… so they could, you know, get the groceries” (FG-PT) were more important to focus on than speed; but, still, “safety is number one” (FG-PT).

### 3.3. Patient Goals and Perspectives of Physical Therapy

When thinking about their most recent episode of physical therapy, people with MS described mostly functional tasks, such as sit to stand, as chief goals. Strengthening of the legs and getting “a good stretching program” (FG-MS) were also frequently desired outcomes. Indeed, several survey respondents specifically described stretching as one of the most effective parts of their physical therapy. Among survey respondents with MS, the most frequently reported goals of therapy were to improve balance (*n* = 32, 78%), improve strength (*n* = 28, 68%), with several participants emphasizing foot drop in particular (*n* = 19, 46%; FG-MS, interview-MS). Related to walking, most people with MS wanted to improve the smoothness and quality of walking (*n* = 28, 68%) and to be able to walk further/longer without needing a rest (*n* = 18, 44%). Increasing walking speed was not a commonly stated goal of physical therapy for people with MS (*n* = 10, 24%).

People with MS expressed satisfaction with physical therapy when they felt heard by their therapist and when they had one-on-one attention, as illustrated by these statements:

“They took the time to listen to me and actually walk with me for a distance to see how my foot/leg behave.” (survey-MS).

“My therapist paying attention and always doing what was best.” (survey-MS).

“It was a waste of time with the last physical therapist I was working with, because they didn't listen to me… They didn't listen to me, so I didn't feel they were working on the things I needed.” (FG-MS).

“But most of the time, I have to say that the physical therapists I've worked with it's the one-on-one for that 15 minutes or whatever it is. You have their undivided attention, and that makes all the different in the world. Don't tell me, ‘Here, go on the New Step for 10 minutes and I'll be back.' I don't want to hear that, you know. You need to be there and find out how I'm ticking.” (FG-MS).

Consistent with this perspective that effective physical therapy involves direct attention from the physical therapist, a couple of survey respondents said that riding a stationary exercise bicycle was the least effective aspect of their physical therapy. Additionally, people with MS are unsatisfied and frustrated when given exercises “that do not work for me” (survey-MS), that are too difficult, or require machines that the person does not have access to without a gym membership.

There was an admission by the focus group participants with MS that they are not always compliant with their home exercises, which was associated with “guilt, because I never do my routine at home” (FG-MS), whereas another participant suggested that noncompliance was related to lack of “incentive to do it when you feel like it's not budging at all” (interview-MS).

It was evident that people with MS also value a therapist who has expertise in MS, commenting that those who are trained in MS “know not to get you to overdo it; they understand MS patients” (FG-MS), whereas, “you know if they are not trained in MS, they want to push, push you, push you and it's like, ‘No, that's not what you do with MS patients.'” (FG-MS). The clinician focus group corroborated this idea, with one therapist stating that, “I have people that are thrilled to have a therapist who has a little bit more appreciation for what MS can be” (FG-PT).

### 3.4. Physical Therapy Treatment Activities and Outcome Measures

Regarding intervention for mobility limitations in MS, the overwhelming theme to arise from the physical therapists was that treatment is a multidimensional approach that includes “a lot of stuff” (FG-PT) and is “as individualized as possible” (survey-PT). The therapists emphasized that it should include “functional activities… rather than giving someone just an exercise to do, make it something that's more important to their life” (FG-PT) or focus on “what's most pressing to them” (FG-PT).

The most frequently reported activities for treatment of walking difficulties were lower extremity strengthening, gait training, and dynamic balance training “because (balance) does impact walking so much” (FG-PT). Therapists also reported focusing on fatigue and spasticity management. Some therapists described using functional electrical stimulation and orthotic devices to help improve walking. The emphases on employing a variety of treatment activities and using a patient-centered approach were consistent across the focus group, interview, and surveys.

Therapists were also in general agreement about the assessment tools used to measure and evaluate walking limitations in MS, with a clear perception of a need to “find something functional and objective to measure” (FG-PT). Thus, the selection of the particular measures used was predominantly based on the patient-specific problems as opposed to routine selection or tests required by the facility. The top responses among survey respondents were the 6-minute walk test (*n* = 11, 69%), fatigue (*n* = 8, 50%), usually assessed with the modified fatigue impact scale or the fatigue severity scale; and the 12-item MS walking scale (*n* = 7, 44%). The timed 25-foot walk test was also reported to be commonly used among all participants, even though “I don't usually work on making them walk faster” (FG-PT), but “usually more just because I feel like I'm lacking in objective measures to say, ‘how is their walking quality'” (FG-PT). Other frequently mentioned outcome measures were the timed up and go test, the functional gait assessment, the dynamic gait index, the Berg balance scale, and the BESTest or the mini-BESTest.

Safety, which was the “number one” walking-related issue among the focus group clinicians, was not spontaneously mentioned during the discussion related to assessment. Thus, the focus group of physical therapists were probed specifically to discuss how they measure safety. After a brief silence and darting gaze to other participants, the following dialogue ensued:

PT6: Mm, it's very subjective. (Group laughs.) I mean, I look, I… (PT2 interjects).

PT2: It's a balance and… (pauses, PT6 continues).

PT6: …I do a lot a lot of questioning, yeah. Loss of balance and, um, I do a lot of questioning on, um, like their insight. Like asking them how they felt they did, during, you know, whatever the gait trial was that we did. Like were they unable to navigate obstacles? Were they steady or unsteady? Are they at risk for falling? Are they walking fast enough to get from point A to point B safely?

PT2: Yeah, and balance confidence is linked with balance ability. (PT6 verbal and non-verbal affirmation.) So balance, you know, so in that way, maybe looking at balance confidence.

PT6: Yeah.

PT5: Yeah, the ABC (Activities-specific Balance Confidence) scale is what I've used before. Just the subjective measure for balance confidence.

PT4: Just whatever balance thing I, whatever balance test I did.

PT5: Measurement (interjecting).

PT4: I guess I only think really of falls when I think of safely walking, but maybe that's very narrow-minded.

PT2: Yeah, I think the loss of balance and falls, how much assistance they need.

PT4: Yeah.

PT2: And those are things that you can measure, count.

PT6: Yeah, their history of falls and I typically ask in the last three months or in the last six months, how many falls they have had.

PT1: Yeah, and I use that too for goal setting. If someone's like, ‘I fall at least two times a week.' Then I might make a goal that they will not fall at all in the span of a week, or something like that.

The verbal and nonverbal expressions suggested a relative lack of certainty about measurement of safety. As the discussion continued, the clinicians described the subjective nature of measuring safety during walking to include observations of “unsteadiness,” “loss of balance,” “frequent toe catching,” and “increased lateral sway.” They also described using “cut off scores” on balance measurement tools to determine fall risk as an indication of a patient's safety.

### 3.5. Assessing Treatment Effectiveness

In terms of knowing if physical therapy has been effective, participants with MS gauged effectiveness by whether “I can do a little bit more than I used to” (FG-MS) and “whether I feel like I can get around more easily and if I don't get tired out as much” (interview-MS). Among the focus group of people with MS, there was unanimous agreement that “not getting worse” was a positive outcome. Indeed, the focus group participants with MS acknowledged that “you're not going to suddenly get better,” and that “this is a disease you live with.” Another person with progressive MS commented that, “I don't have any great expectations at this point, but I would like to be able to keep at least doing what I can do” (interview-MS).

This finding was strongly corroborated by the survey responses in which, “I didn't get any worse during the therapy period” (*n* = 14, 34%) was the most frequently selected response option to indicate that physical therapy was effective. Other common responses included, I am more confident in my abilities (*n* = 13, 32%), I am not as wobbly or unsteady when I walk (*n* = 11, 27%), and I feel like I can do more (*n* = 11, 27%); the latter also being consistent with the focus group and interview findings. Importantly, “I can walk faster” was not a frequently reported indicator of therapeutic effectiveness among survey respondents with MS (*n* = 6, 15%). Indeed, the “most important” to “least important” aspects of mobility as ranked by survey participants with MS were as follows:

1. I do not want to fall
2. I want to walk without an assistive device
3. I want to be able to walk more smoothly
4. I want to be able to walk longer distances
5. I want to walk faster
6. I want to be able to talk or do other things while walking
7. Something else is more important (examples provided were as follows: “I want to feel more confident in my ability to walk,” “I want to get stronger,” “I need to be able to negotiate stairs,” and “Play with my kids”)

For physical therapy clinicians, determining effectiveness of treatment is primarily based on improvement in outcome measures at reassessment. There was a strong consensus from all clinician participants, as exemplified by these excerpts:

“I guess this is obvious, but when we do our assessments, fortunately or unfortunately, it's very based on all these outcome measures we just talked about.” (FG-PT, non-verbal affirmation from other participants).

“Well, of course, repeating the outcome measures and kind of using that as a guiding tool.” (interview-PT).

“If measures improve.” (survey-PT).

“Reassessment of outcome measures comparing at baseline vs re-evaluation.” (survey-PT).

There was also agreement that patient self-report and, where applicable, family report is used to help determine if treatment had helped the patient. For example,

“Report of family, patient's perspective.” (survey-PT).

“But a lot of it, use patient reports of them being able to participate in different activities that they weren't able to do before. I've also found that family members and other caregivers, their input on how the person's performing their day-to-day activities, walking, how far they'll be able to walk, that subjective report from caregivers…” (interview-PT).

“I think using some of those self-report measures is a great way to do that (know if a patient has benefited from therapy). Besides just asking, ‘Hey, how are you feeling?' from session to session, but when you're doing an actual assessment, to say, ‘Let's do the ABC scale again or the MS Fatigue Impact or the walking scale.'” (FG-PT).

The idea that maintenance of functional level can be important also came up in the clinician focus group, with one therapist commenting:

“And I think a challenge for patients that have MS is also just the progressive nature of the condition. And where they are along that progression because some people progress much more rapidly than others. So, a lot of times you're looking to see if you've made improvements, whereas other times it's a victory to have maintained where they were, when you first originally started working with them.” (FG-PT).

The consensus on this idea was not as strong among the clinicians as it was among the focus group of people with MS. Further, it was clear that even though therapists recognize that patients with MS have a progressive disease, they like to give the patients activities or home exercises that make the person feel as though they have been “productive and successful” (FG-PT). Indeed, most survey respondents indicated that they were looking for patients to report “improved function.” None of the clinician survey respondents mentioned maintaining function as a demonstration of treatment efficacy. However, this may be because the questions directed them to think about a particular bout of physical therapy, rather than long-term management of patients.

## 4. Discussion

The purpose of this study was to identify the most important aspects of mobility for people with MS and physical therapy clinicians experienced in treating MS-related mobility impairments and to explore how patients and clinicians perceive whether physical therapy has been effective. The findings revealed that the most important priority for people living with MS was safety/avoiding falls. People with MS also regarded being able to participate in activities outside the home as highly important. Walking speed was not a high priority for people living with MS, with many participants acknowledging that slowness was a deliberate strategy for maintaining safe ambulation. Indeed, several other aspects of mobility were considered by patients with MS to be more important than walking speed, including not needing to rely on an assistive device, smoothness of walking quality, and endurance/distance.

Safety was also the most important aspect of mobility expressed by clinicians. Although the clinicians described routinely measuring walking speed as part of their evaluation, the clinical decision-making process appeared to be driven by the need to document objective measures, as opposed to setting walking speed as a treatment goal. Walking speed assessments were also often used by clinicians to make inferences about safe and functional ambulation in the community. Indeed, gait speed is a well-recognized indicator of functional status and health [23]; thus, its frequent use by clinicians as part of the evaluation profile is warranted even though it may not be the primary intervention target.

Interestingly, although there is evidence that “fast” walking speed in people with MS can be improved following rehabilitation or exercise interventions in both relapsing-remitting [24–27] and progressive forms of MS [28–32], self-selected walking speed does not necessarily increase despite the significantly improved capacity to walk faster [28]. Thus, individuals with MS may choose not to walk faster, even though the capacity to do so may be present. This observation is consistent with our findings that walking quickly is not an important priority for people with MS.

Although safety was the “number one” priority for clinicians, there was some uncertainty regarding practices to objectively measure improvements in safety. Rather, it appeared that clinicians relied on a subjective impression of safety informed by observations of stability or losses of balance during mobility-related activities, as well as consideration of patient-reported fall history. Clinicians described using standardized balance assessments with established associations with fall risk to objectively measure constructs related to patient safety. Indeed, given the focus of both patients and clinicians on the importance of avoiding falls, measures of dynamic balance and reactive postural control may be more meaningful than gait speed for evaluating therapeutic effectiveness of rehabilitation interventions for walking limitations. This is significant because a recent systematic review of outcome measures used in trials of gait rehabilitation in MS [33] found that 87% of trials reported gait speed as an outcome and that gait speed was rarely combined with measures of balance (19% of studies). Thus, current clinical trial outcomes are not well aligned with patient priorities, based on the findings from our study.

Regarding how patients and clinicians evaluate treatment efficacy, there were some incongruencies between the two perspectives. In particular, many people living with MS reported that “not getting worse” is a successful outcome from an episode of physical therapy, whereas clinicians expected improvements in outcome measures exceeding minimal clinically important differences to demonstrate efficacy. However, clinicians also reported relying on patient and caregiver report of improved function. Interestingly, the systematic review of outcome measures used in trials of gait rehabilitation in MS [33] found that self-reported measures are not often used to evaluate outcomes in clinical trials. The comments by people living with MS that maintaining current functional level (i.e., not getting worse) is an important indicator of treatment effectiveness, although unanimous in the focus group, were not unanimous across all respondents. A small number of participants with MS in the focus group reported feeling frustrated when exercises do not help them or they “didn't get any better” with physical therapy. Thus, while some people with MS may be satisfied with not declining, other people with MS maintain a degree of expectation that they can improve their function with targeted rehabilitation. Differences in expectations may be related to current functional status or disease phenotype (e.g., a person in remission versus a person with advanced or progressive disease) and previous experiences with physical therapy. Regardless, a patient's expectations may influence their perceived response to rehabilitation and should be considered when mutually establishing treatment goals with the clinician.

A limitation of this study was the limited number of focus groups that could be conducted due to complexities of participant availability and geographic location. However, the focus groups and one-on-one interviews informed the design of the survey, which provided valuable triangulation of the data. The questions in the focus groups, interviews, and surveys did not go into depth regarding the role of fatigue in walking difficulty in MS. Because fatigue can substantially limit mobility in MS [34], it may be an important component of mobility evaluation in rehabilitation practice and research and could potentially impact perceived or actual responsiveness. The participants with MS were quite heterogenous, and although our goal was to obtain a diverse range of opinions and experiences from people with all types of MS, in the future, it may be useful to use segregation of focus groups to explore if there are differences in experiences and perspectives between people with different types of MS. The study was conducted in the United States of America; thus, the perspectives of patients and clinicians identified here may not be applicable to clinicians and patients in other health care systems. It is also important to disclose that all of the authors/researchers are licensed physical therapists with expertise in multiple sclerosis; thus, the interpretation of the data and conclusions were grounded in their specific set of expertise and experiences.

## 5. Conclusions

This qualitative study revealed that walking speed, despite its recognized functional significance in the clinical community and popular use in evaluating rehabilitation efficacy in clinical trials, is not of prime importance to people living with MS or to clinicians treating MS-related mobility impairments. Clinicians and patients were congruent in their prioritization of fall avoidance and safety but were somewhat incongruent in how they evaluate whether a bout of physical therapy has been effective. Given that patient perception of improvement is central to clinical significance, clinicians and researchers should consider incorporating assessments of dynamic postural control and fall risk when evaluating efficacy of interventions targeting mobility disability in MS.

## Data Availability

The qualitative data used to support the findings of this study are available from the corresponding author upon request.
